# Spinal plasticity in robot-mediated therapy for the lower limbs

**DOI:** 10.1186/s12984-015-0073-x

**Published:** 2015-09-17

**Authors:** Andrew JT Stevenson, Natalie Mrachacz-Kersting, Edwin van Asseldonk, Duncan L. Turner, Erika G. Spaich

**Affiliations:** Center for Sensory-Motor Interaction (SMI), Department of Health Science and Technology, Aalborg University, Fredrik Bajers Vej 7 D-3, Aalborg, DK 9220 Denmark; Biomedical Engineering, University of Twente, 7522NB Enschede, The Netherlands; NeuroRehabilitation Unit, School of Health, Sport and Bioscience, University of East London, London, E15 4LZ England

## Abstract

Robot-mediated therapy can help improve walking ability in patients following injuries to the central nervous system. However, the efficacy of this treatment varies between patients, and evidence for the mechanisms underlying functional improvements in humans is poor, particularly in terms of neural changes in the spinal cord. Here, we review the recent literature on spinal plasticity induced by robotic-based training in humans and propose recommendations for the measurement of spinal plasticity using robotic devices. Evidence for spinal plasticity in humans following robotic training is limited to the lower limbs. Body weight-supported (BWS) robotic-assisted step training of patients with spinal cord injury (SCI) or stroke patients has been shown to lead to changes in the amplitude and phase modulation of spinal reflex pathways elicited by electrical stimulation or joint rotations. Of particular importance is the finding that, among other changes to the spinal reflex circuitries, BWS robotic-assisted step training in SCI patients resulted in the re-emergence of a physiological phase modulation of the soleus H-reflex during walking. Stretch reflexes elicited by joint rotations constitute a tool of interest to probe spinal circuitry since the technology necessary to produce these perturbations could be integrated as a natural part of robotic devices. Presently, ad-hoc devices with an actuator capable of producing perturbations powerful enough to elicit the reflex are available but are not part of robotic devices used for training purposes. A further development of robotic devices that include the technology to elicit stretch reflexes would allow for the spinal circuitry to be routinely tested as a part of the training and evaluation protocols.

## Introduction

Robot-mediated therapy is utilized to restore motor function in patients with central nervous system damage. One application for robotic devices is aimed at improving walking ability in patients, particularly following either spinal cord injury (SCI) or brain injury. Robotic devices allow for the body weight of patients to be supported and enable mobilization of the joints when patients cannot achieve this without aid. Robotic exoskeleton devices have been employed in numerous studies and have a positive effect in improving walking ability in SCI and stroke patients [1–5]. Until recently, however, few studies have investigated the underlying neural mechanisms for the functional improvements observed following robot-mediated therapy in humans, particularly within the spinal neural circuitry. A greater focus on spinal plasticity following robotic training to improve gait after neurological injuries may help to target and refine the rehabilitation strategies employed by therapists and establish neurophysiological measures of activity-dependent plasticity that predict recovery of motor function.

The major objectives of this review are to a) review the recent literature on spinal plasticity induced by robot-mediated therapy in humans and b) propose recommendations for the measurement of spinal plasticity using robotic devices in order to increase our understanding of the neural mechanisms underlying functional recovery in patients and improve robot-mediated therapy protocols. This work was developed in the frame of the project “STate of the Art Robot-Supported assessments” (STARS) as part of the COST Action TD1006 “European Network on Robotics for NeuroRehabilitation” [6]. STARS is intended to serve clinical practitioners, technology developers and manufacturers, and scientists active in the field of neurorehabilitation. The goal is to give recommendations for development, implementation, and administration of different types of robot-mediated assessments of motor function, grounded on the current scientific literature.

A recent review on robotic training and spinal plasticity by Edgerton and Roy [7] highlighted that in both human and animals the spinal cord has the potential to reorganize and/or readjust to the loss of supraspinal input and utilize the remaining peripheral (afferent) input to control stepping and standing. However, the major focus of the review by Edgerton and Roy [7] was on animal studies, since no studies existed at the time specifically investigating spinal plasticity in humans following robotic training. To our knowledge, there is no direct evidence for spinal plasticity following robot-mediated therapy in the upper limbs. Only one study to date has investigated spinal plasticity in the upper limbs in hemiplegic stroke patients, which found that following a standardized passive exercise program using a robotic arm, there were no significant alterations in the H_max_, M_max_ or H_max_/M_max_ ratio as compared to pre-exercise evoked responses [8]. Additionally, healthy individuals show no changes in spinal reflex pathways in the lower limbs following body weight supported (BWS) robotic-assisted step training [9, 10]. However, spinal reflex pathways are phase modulated throughout the gait cycle during BWS robotic-assisted step training in a similar manner to unassisted walking [11].

Recent patient studies have demonstrated a number of plastic changes in spinal pathways following BWS robotic-assisted step training on a motorized treadmill [12–18]. Hemiplegic stroke patients showed changes in postactivation depression, as assessed by frequency-related depression of the soleus H-reflex, following robotic-assisted gait training [18]. Postactivation depression is a presynaptic mechanism regulating the excitability of the stretch reflex, and is decreased in patients with spasticity [19, 20]. Following four weeks (12 sessions) of BWS robotic-assisted gait training, hemiplegic patients with spasticity showed a normalization of postactivation depression. Changes were also significant following just one training session [18].

While evidence for spinal plasticity in stroke patients is limited to the study by Trompetto et al. [18], SCI patients training using BWS robotic-assisted step training also exhibited changes in neuromuscular function. These include: a) changes in amplitude and phase modulation of short-and long latency withdrawal reflexes elicited by electrical stimulation of a cutaneous nerve during walking; b) a re-established physiological phase modulation of the soleus H-reflex during walking; c) increased presynaptic inhibitory control of soleus motoneurons assessed by conditioning the soleus H-reflex with electrical stimulation of the common peroneal nerve, d) changes in action of Ia and Ib inhibitory interneurons acting on soleus motoneurons at rest and during locomotion, assessed by conditioning the soleus H-reflex with electrical stimulation of the common peroneal nerve or the medialis gastrocnemius nerve; e) changes in contraction levels between knee and ankle antagonistic muscles and finally f) an improved intra- and interlimb muscle coordination [12–16]. Furthermore, BWS robotic-assisted step training also decreased biomechanical measures of reflexive joint stiffness in the ankle of SCI patients [17]. Given that some of the individual SCI patients in these studies had either motor complete or sensory-motor complete SCIs, it is likely that the plasticity in the spinal cord occurred with little or no descending supraspinal input [14]. These findings support the notion that spinal neuronal networks of patients with clinically complete, motor complete, or motor incomplete SCI have the potential to undergo a reorganization of spinal neural circuits, improve spinal reflex function, and improve walking function with BWS robotic-assisted step training.

The most notable sign of spinal plasticity following BWS robotic-assisted step training in SCI patients was the re-emergence of walking phase modulation of the soleus H-reflex [12], which can be viewed as a net plasticity of multiple spinal networks [14]. The patients tested in this study had chronic clinically complete, motor complete, and motor incomplete SCI and received an average of 45 training sessions, five days per week for one hour per day. Following training, the soleus H-reflex was depressed at late stance, stance-to-swing transition, and swing phase initiation, allowing for a smooth transition from the stance to swing phase [12]. The soleus H-reflex remained depressed at early and mid-swing phases of the gait cycle, which fostered a reciprocal activation of the ankle flexors and extensors. The training reorganized spinal locomotor neural networks, evoking a functional amplitude modulation of the soleus H-reflex and therefore a more natural step progression. Moreover, the BWS robotic-assisted step training altered the amplitude and onset of muscle activity during walking, decreased step duration, and improved the gait speed [12].

While it was concluded that the primary source of the neuronal plasticity observed in these studies is located in spinal locomotor networks, plasticity of supraspinal connections with spinal locomotor networks may also be altered [12]. However, regardless of the precise location of neuronal plasticity, these studies show that patients with clinically complete, motor complete, or motor incomplete SCI have the potential for an adaptive reorganization of spinal neural pathways and improved walking function following BWS robotic-assisted step training [12–16]. A limitation of the studies by Knikou’s group is that the number of patients were relatively low, with data from only one patient with American Spinal Injury Association Impairment Scale (AIS) A and one patient with AIS B, and low sample sizes of patients with AIS C (*n* = 6) and D (*n* = 8) [14]. Larger scale neurophysiological studies are needed to comprehensively characterize spinal plasticity in patients with different types and levels of SCI. Furthermore, no significant improvements in walking or sit-to-stand ability were observed after the training as assessed by the six-minute walk test, the timed up and go test, and the sit-to-stand test [14]. These results are similar to studies investigating cortical plasticity in patients groups, where there is not necessarily a correlation between cortical plasticity and clinical improvement [21, 22]. However, the lack of relationship between changes in spinal plasticity and functional recovery is also likely due to the low sample sizes and therefore a lack of statistical power, the variable number of training sessions between patients, and also due to the sensitivity to changes in the clinical tests used for assessing improvements in walking function, highlighting the need for further research on this topic [14].

Spinal plasticity was also induced following operant conditioning training of simple spinal reflex pathways for muscles of the lower leg elicited by electrical stimulation [23–27] and a simple robotic actuator device [28–30]. Spinal reflex conditioning can alter the size of spinal reflexes and leads to functionally beneficial alterations in the modulation of reflexes during dynamic activities in both healthy individuals and patients with SCIs [24–27]. Studies in monkeys and rats show that soleus H-reflex conditioning is associated with plasticity at multiple sites in the spinal cord and brain, including changes in motoneuron properties and spinal interneurons [24–27]. While conditioning protocols can target plasticity to specific neural pathways, the benefits of the conditioning appear to be much more widespread than what can be attributed to the specific pathway. For example, unilaterally decreasing the size of the soleus H-reflex in SCI patients led to faster and more symmetrical gait in these patients, as well as an increase in the modulation of muscle activity bilaterally [24, 25, 27]. Therefore, specific conditioning protocols can be designed to target specific deficits in patients [23].

While operant conditioning of spinal reflex pathways elicited by electrical stimulation may be beneficially incorporated into lower limb robot-mediated therapies in order to induce beneficial spinal plasticity in normal, patient, and athlete populations, operant conditioning of the human soleus stretch reflex has been successful using a simple robotic mechanical actuator device. Indeed, soleus spinal stretch reflex pathways can be both increased (up-conditioned) and decreased (down-conditioned) in healthy participants in the same way as soleus H-reflexes using a mechanical actuator system [25]. Following successful soleus stretch reflex up-conditioning in healthy individuals, afferent feedback is enhanced leading to an increase in stiffness around the ankle joint [28–30]. Furthermore, increased soleus stretch reflexes led to a substantial reduction in centre of pressure excursions when landing on one leg following a drop jump from a 30 cm height [29]. Similar reflex conditioning protocols may also benefit elderly or patient populations with an elevated risk of falling [29].

A recent review of robotic therapy for rehabilitation suggests that lower extremity robotic therapies are in their relative infancy compared with upper limb robotic therapy [29]. While a recent meta-analysis including 23 trials with a total of 999 patients showed a small additional value for robot-assisted gait training combined with conventional training compared to conventional training alone, especially for the more severely affected patients, the effectiveness of this form of training could clearly be further improved [31]. With more knowledge about the underlying neurophysiology and spinal plasticity in SCI and brain-injured patients following robotic training assessed via specific neurophysiological techniques, the training may possibly be more targeted for specific patients in order to maximize recovery. Indeed, there is evidence for plasticity in a number of spinal reflex pathways in stroke and SCI patients following BWS robotic-assisted step training, with many spinal pathways becoming more like healthy controls in terms of phase modulation and amplitude during locomotion [12–16, 18]. Furthermore, operant conditioning of soleus stretch reflexes using a simple robotic actuator device provide a promising direction of future research given the stability improvements in healthy subjects and the functional improvements observed in patients following targeted conditioning of the H-reflex [23–30].

### Definition of measure

Alterations in spinal pathways have the advantage that they are directly linked to motor behaviour and as such one may not only quantify the responses electrophysiologically, but also behaviourally (for example, a change in the forces or torques around the target joint) [2, 32]. To date, the majority of studies continue to report reflex response sizes from electromyographic (EMG) data, likely since early reports indicated that the behavioural response varies from one elicited reflex to the next [17, 33, 34]. Regardless of the outcome measure, the importance of the spinal cord from a clinical perspective resides in the fact that it forms the final pathway to the ensuing behaviour. Therefore, the appropriate guidance of spinal cord plasticity likely plays a major role in restoring useful function following a central nervous system insult such as SCI or stroke [35, 36]. However, any alterations at the level of the spinal cord will also have an influence (and on the converse be influenced) by changes in higher-level structures [25, 37]. For example, the operant conditioning protocol mentioned previously not only up and down-regulates the size of the H- and stretch reflex, but also significantly affects more complex behaviors beyond the conditioned pathway such as improvements in walking [25, 37]. Indeed, spinal and supraspinal plasticity underlies operant conditioning [16]. In conclusion, many pathways may be affected by the rehabilitation intervention both within the spinal cord as well as within other areas of the central nervous system and as such, a clear definition of spinal plasticity remains elusive.

The neurophysiological methods that can be used to assess spinal plasticity are based on the utilization of spinal reflexes to probe the spinal networks. These reflexes include for example, the H-reflex, the stretch reflex, and withdrawal reflexes. However it is important to note that all spinal reflexes are under the control from higher centers, being modulated in a task specific manner. Eliciting H-reflexes and withdrawal reflexes require the use of electrical stimulation, which is not a natural part of a robotic device. The technology needed to elicit stretch reflexes could, on the other hand, be integrated in a robot and thereby be used to routinely assess training-induced spinal plasticity. In a clinical setting, typically, tendon taps are applied to investigate where along the spinal cord an injury has occurred. These are then repeated as the rehabilitation program progresses to assess for any changes. While tendon taps are useful tools to reveal the level of a lesion, the inability to reproduce the same tapping force and speed from one trial to the next makes it impossible to be implemented in a reliable manner. For this purpose, when eliciting the stretch reflex, it is necessary to use tools that allow a precise extension of the muscle or tendon such as an actuator system that imposes joint rotations [for review, please see 38]. In this sense, rehabilitation robots should have the ability to induce such joint rotations. Stretch reflexes elicited by joint rotations have the additional benefit that they result in a more natural, asynchronous input to the central nervous system, directly activating muscle afferent receptors. Furthermore, stretch reflexes also change with targeted training [28–30].

### What to measure

The following elements should be quantified when assessing spinal plasticity utilizing the stretch reflex:

1. The stretch reflex size in terms of a) the EMG response in a defined window following the stretch onset with regards to the baseline EMG and b) the force or torque produced around the respective joint. Assessing the changes in the amplitude of stretch reflexes offers an objective method for measuring spinal plasticity in the central nervous system provided they are due to neural effects and not changes in the state of the muscle. The stretch reflex can be typically separated into two or three clearly defined peaks [39, 40]. The initial short-latency response represents the monosynaptic excitation of motoneurons in the spinal cord [41], while the middle-latency component is likely mediated by group II afferents [42]. In contrast, a transcortical pathway likely contributes to the long-latency component [40, 43–46]. Measuring the amplitude of the short-latency response would therefore be of relevance to assess spinal plasticity.
2. Latency of the onset of the stretch reflex both from EMG and from the force or torque produced around the joint, particularly of the short-latency component.
3. The active and passive phases of the imposed stretch. These phases relate to the initial rotation of the joint by the mechanical actuator (active) and the hold phase of the perturbation (passive; typically around 200 ms) before the joint is released [47–49]. The active phase of the imposed stretch, causing the initial change in joint angle and therefore muscle length, is the most important in relation to the assessment of spinal plasticity.
4. When possible, the measurement should be provided during active functional movements such as walking or standing.
5. Further, using the soleus muscle as a model, if possible, is useful since the majority of previous studies have investigated this muscle both during static and dynamic tasks and thus normative data is available for both healthy subjects and spastic patients [49, 50].
6. However, where possible other muscles such as the tibialis anterior, the quadriceps and hamstring muscle group may also be investigated due to their relevance to human gait.

### Systems for measurement

To assess stretch reflex responses, perturbations need to be applied to a joint. The main requirement for these perturbations is that they need to be fast enough to trigger a reflex. For healthy individuals, for instance, the required velocities to trigger a stretch reflex response in the knee or ankle extensors are in the range of 230–360 deg/s [e.g. 47, 51]. Robotic devices have the potential to provide these perturbations. However, the required velocities and accompanying accelerations put high demands on the actuation and mechanical linkage of the device. The actuators need to be powerful and the linkage needs to be lightweight to prevent that a large amount of torque is “wasted” to accelerating the inertia of mechanical linkage. To the authors’ knowledge, there is no lower limb robotic trainer available, either commercially or in academia that is capable of providing such fast perturbations. For example, BWS robotic-assisted step trainers have been used once recently to elicit reflex responses [52]. However, the induced responses generally occurred after about 100 ms and could therefore not be regarded as monosynaptic spinally mediated stretch reflexes [40, 50, 52]. Only dedicated experimental devices are currently able to provide these perturbations during gait [47, 51]. Generally, these devices have powerful actuators detached from the exoskeleton and positioned remotely and use a flexible transmission to transfer the forces/torques to a lightweight exoskeleton attached to the subject’s joint.

In principle, force and position perturbations can be used to elicit the reflex response. Still, the aforementioned devices all use position perturbations [47, 51]. As stated before, the reflex magnitude to position perturbations can be quantified from recorded EMG responses and/or interaction forces. Although measuring the EMG responses allows identifying hyperreflexia, it does not provide information on the resistance to movements (reflexive stiffness) resulting from these increased reflexes. To determine the reflexive stiffness, complex system identification techniques are required. These techniques have been used to assess the reflexive stiffness in quasi-static situations and different groups have shown that the reflexive stiffness is increased in different populations of neurological patients [53, 54]. However, they have not been used to quantify the reflexive stiffness in response to perturbations during walking. This would require further extension of these techniques and foremost a device that is capable of providing fast perturbations while reliably measuring the interaction forces [47, 51].

### What is normal?

In healthy subjects, the short-latency soleus stretch reflex has been shown to be phase-modulated during gait. In the stance phase, the amplitude was similar to that measured during standing at matched soleus background EMG; in the transition from stance to swing, the amplitude was zero, while in late swing, the amplitude was 45 ± 27 % of the maximal amplitude in the stance phase (34 ± 9.3 μV) for a stretch amplitude of 8° and a stretch velocity of 25O deg/s [49]. The mid- and long-latency stretch reflexes were similarly modulated [50]. The onset of the short-, mid-, and long- latency stretch reflex (56.0 ± 0.7 ms, 84.9 ± 1.3 ms, and 113.9 ± 3.4 ms, respectively) did not depend on the phase in the step cycle where it was elicited [50]. The stretch reflex response in the extensors of the knee of healthy subjects was also modulated in the transition from swing to stance; the magnitude, expressed as a percentage of the maximum absolute stretch reflex magnitude, peaked at 15–20 % of the gait cycle and was approximately 35 % of the maximum stretch reflex magnitude during the late swing phase [48].

### Recommendations for measurement

When using robotic devices to quantify the stretch reflex size, several important considerations must be made:

1. Since spinal plasticity may cause either an up or a down-regulation of the stretch-reflex size, the parameters of the imposed stretch must be adjusted to allow for the reflex to be either increased or decreased compared to the previous measurement. The parameters should thus be set such that there is no saturation of the motoneuronal pool, nor should these cause a just above threshold response.
2. Patients may also suffer from spasticity, which is a confounding factor when assessing spinal plasticity using the stretch reflex. It is not possible to avoid a spastic reaction if a patient is indeed spastic when using the stretch reflex as an assessment tool. However, if the same stretch parameters are used over the course of a rehabilitation program then the assessment may reveal a change in the spastic behaviour as has been shown following H-reflex conditioning in spinal cord injured patients [23].
3. Patients may only be able to walk for short periods of time. However, for accurate representation of the reflex responses, at least 10–30 reflexes should be averaged [48–50] together with control trials where no reflex is elicited. This will consequently limit the amount of time points in which the reflex would be elicited during the gait cycle to investigate the phase modulation of the stretch reflex.

## Conclusions

The evidence surrounding plastic changes in spinal pathways following robot-assisted training is scarce and limited to the lower limbs; however, many spinal reflex pathways in stroke and spinal cord injured patients behave more like those of healthy controls following robot-mediated training. Among the techniques used to assess these changes, stretch reflexes elicited by mechanical perturbations of different joints are of special interest since the actuator system necessary to elicit the reflex could be naturally integrated into robot devices. Assessing training-induced spinal plasticity could therefore be a part of the training and evaluation protocols, further increasing our understanding of the neural mechanisms underlying functional recovery in patients and allowing for the possibility that training could be more specifically targeted to maximize recovery.
